# Safety and efficacy of long-term use of sodium oxybate for narcolepsy with cataplexy in routine clinical practice

**DOI:** 10.1016/j.sleep.2017.03.028

**Published:** 2017-07

**Authors:** Panagis Drakatos, Dimosthenis Lykouras, Grainne D'Ancona, Sean Higgins, Nadia Gildeh, Raluca Macavei, Ivana Rosenzweig, Joerg Steier, Adrian J. Williams, Rexford Muza, Brian D. Kent, Guy Leschziner

**Affiliations:** a Sleep Disorders Centre, Guy's Hospital, Great Maze Pond, London, SE1 9RT, United Kingdom; b Department of Pulmonary Medicine, University Hospital of Patras, Rio Patras 26500, Greece; c Sleep and Brain Plasticity Centre, Department of Neuroimaging, IoPPN, King's College London, United Kingdom; d Faculty of Life Sciences and Medicine, King's College London, United Kingdom; e Department of Basic and Clinical Neuroscience, King's College London, United Kingdom

**Keywords:** Sodium oxybate, Narcolepsy, Cataplexy, Adverse effects

## Abstract

**Background:**

Sodium oxybate is licensed in Europe for the treatment of narcolepsy with cataplexy in adults. The aim of this study was to assess the efficacy and safety of sodium oxybate in clinical practice in patients with narcolepsy and cataplexy refractory to other treatments.

**Materials and methods:**

This was a retrospective single centre study including patients with severe narcolepsy with cataplexy refractory to other treatments, who were initiated on sodium oxybate between 2009 and 2015. Patients were allowed to be on other stimulants or/and anti-cataplectic agents. Epworth sleepiness scale (ESS) and weekly cataplexy events were recorded. Side effects (SEs) were recorded at every follow-up visit.

**Results:**

90 patients were prescribed sodium oxybate, with a total of 3116 patient-months of drug exposure. ESS and weekly cataplexy events were significantly reduced by sodium oxybate for all patients (ΔESS = 4.3 ± 4.4 and Δcataplexy = 21.8 ± 18.5 events/week; p < 0.0001, respectively). The required maintenance dose could not be predicted based upon gender, body mass index, or clinical factors. 60% of patients were able to reduce or come off other medications. Half of the patients experienced at least one SE, and 26.6% had to stop treatment due to limiting SEs. Nausea, mood swings and enuresis were the most commonly reported SEs. SEs that led to drug discontinuation, particularly psychosis, were associated with increasing age and were observed early after the initiation of the drug.

**Conclusions:**

Sodium oxybate provides a good clinical efficacy and acceptable safety profile in routine clinical practice for the treatment of patients suffering from narcolepsy with cataplexy. A quarter of patients experience SEs requiring withdrawal of the drug with older patients being more vulnerable to the more serious SEs.

## Highlights

• Sodium oxybate improves alertness in refractory to treatment patients with narcolepsy with cataplexy.
• Sodium oxybate improves cataplexy in refractory to treatment patients with narcolepsy with cataplexy.
• 1/4 of the patients had to withdraw from the drug due to side effects.
• In half of the patients sodium oxybate led to reduction of other drug dosages and in 10% to discontinuation.

## 1 Introduction

Narcolepsy is a chronic neurological disorder causing dysregulation of sleep and wakefulness, with a prevalence of approximately 1 in 3000 [1]. It is characterised by excessive daytime sleepiness (EDS), cataplexy, hypnagogic/hypnopompic hallucinations, sleep paralysis and disturbed sleep [2].

There is no cure for narcolepsy at present, and current treatment focuses on symptom control. A reduction in daytime sleepiness is achievable with medications that increase wakefulness, including wake promoters such as modafinil, amphetamines, and methylphenidate, while cataplexy is typically controlled through the use of antidepressant drugs which suppress Rapid Eye Movement sleep [3], [4], [5], [6].

Sodium oxybate, the sodium salt of gamma-hydroxybutyrate (GHB), an endogenous metabolite of gamma-aminobutyric acid (GABA), is a central nervous system depressant. It is indicated for the treatment of both cataplexy and EDS associated with narcolepsy [7]. Its mode of action is uncertain, but it may involve stimulation of GABA-B receptors [8]. Sodium oxybate is rapidly absorbed and metabolised, having a mean elimination half-life of 90–120 min. It is currently authorized by the European Medicines Agency to treat narcolepsy with cataplexy in adults, and by the US Food and Drug Administration (FDA) to treat cataplexy in patients with narcolepsy, with an “expanded indication” for the treatment of EDS [3], [9].

Existing data regarding the tolerability and efficacy of sodium oxybate in narcolepsy originate from prospective drug trials [10], [11], [12], [13]. We aimed to analyse the use of sodium oxybate in routine European clinical practice, where sodium oxybate is reserved for a sub-group of patients with narcolepsy with cataplexy, patients more likely to be on multiple other therapies with a more severe phenotype.

## 2 Materials and methods

We performed a retrospective study assessing patients attending a tertiary referral sleep disorders centre. All patients diagnosed with narcolepsy with cataplexy and initiated on sodium oxybate between 2009 and 2015 were included. A full medical history, sleep history, sleep study results and demographics were recorded for all patients. The reduction of Epworth Sleepiness Scale (ESS) score (ΔESS) and of cataplexy events expressed in events/week (Δcataplexy) were recorded from patients' notes and relevant diaries, as the differences between ESS and cataplexy events before sodium oxybate initiation (baseline) and after a dose of sodium oxybate providing optimal clinical effect was achieved (final).

Due to the nature of the study and the limitations that apply in the UK for sodium oxybate treatment eligibility, patients could already be on other drugs for narcolepsy (stimulants or/and anti-cataplectic agents). According to policies in place for prescribing of sodium oxybate within the UK National Health Service (NHS), patients were only eligible for this drug if they had tried at least two stimulants and/or two anti-cataplectic agents, and remained symptomatic with an ESS ≥17 or with an average 21 cataplectic events per week.

All the patients in this study were initially treated with oral sodium oxybate 4.5 g/night, given as two equally divided doses 2.5–4 h apart and titrated, according to response, up to a maximum dose of up to 9 g/night in two doses of 4.5 g each, with dose adjustments every two weeks, as stipulated in the approved prescribing protocol. Any subsequent reduction or elimination of other drugs for narcolepsy was recorded. Appropriate approval from the institutional review board on human research was obtained (project number 4641).

## 3 Diagnosis

The diagnosis of narcolepsy with cataplexy was made according to the International Classification of Sleep Disorders-2 (ICSD-2) criteria of the American Academy of Sleep Medicine (AASM), due to the retrospective nature of the study [14].

## 4 Outcomes analysis

Safety and tolerability were evaluated based on reported side effects (SEs), without a pre-established list of SEs, during every follow-up visit after initiation of sodium oxybate. SEs were categorized into groups: namely infections, psychiatric, neurological, gastrointestinal, general, sleep disorders and cardiological SEs, in keeping with those listed in the summary of product characteristics [15]. Reports from patients' notes of mood changes throughout the day, which could vary from elevated mood to anger to sadness within a few hours, and changes in mood clearly out of proportion to circumstances which could also cause impairment in functioning, were grouped under the term mood swings. Psychotic symptoms were confirmed by formal psychiatric assessment and were made according to the Diagnostic and Statistical Manual of Mental Disorders fifth edition (DSM-V) criteria [16].

## 5 Statistics

Results are reported as mean ± SD if not otherwise indicated. Following testing for normality, comparison between groups was performed using independent-samples Mann–Whitney *U* test. Spearman product correlation coefficient was used for correlations between continuous and nominal variables and Chi Square with Cramer's V product for correlations between nominal variables. Comparison of baseline and final ESS and cataplexy events within patients was calculated using related-samples Wilcoxon Signed-Ranks Test. A multiple stepwise linear regression analysis was performed to identify which variables most satisfactorily explained ΔESS and Δcataplexy in our patients. Binary regression analysis with forward stepwise method was applied in three prediction models: 1) to predict which patients exhibited a reduction of other drugs (wake promoters, stimulants or/and anti-cataplectic agents) due to sodium oxybate initiation, 2) which experienced any SEs secondary to sodium oxybate, and 3) which patients had to discontinue the drug due to SEs. Statistical significance was defined as p < 0.05. IBM SPSS Statistics V24.0 (SPSS, Chicago, IL/USA) was used for all statistical analysis.

## 6 Results

### 6.1 Efficacy

A total of 90 patients with narcolepsy with cataplexy, aged 42.5 ± 14.9 years, were prescribed sodium oxybate during the study period, with a total of 3116 patient-months of drug exposure, and a median 35.5 months (interquartile range 11.0–54.0) of follow up time (FU) (Table 1).

**Table 1:** Demographics, baseline characteristics and treatment of the studied cohort.

Parameters (n = 90)	Mean ± SD
Age (years)	42.5 ± 14.9
Gender (M/F)	38/52
BMI (kg/m^2^)	30.1 ± 7.8
*ESS baseline	18.9 ± 3.4
*ESS final	14.5 ± 5.1
*Cataplexy events/week baseline	26.2 ± 22.7
*Cataplexy events/week final	4.4 ± 10.8
Patients on stimulants or wake promoters, n (%)	36 (40)
Patients on anticataplectic agents, n (%)	7 (7.8)
Patients on combination treatment, n (%)	44 (48.9)
Patients on no treatment, n (%)	3 (3.3)
FU time (months)	35.5 (IQR 11.0–54.0)

BMI, Body Mass Index; ESS, Epworth Sleepiness Scale score. *: Baseline and final term uses as reference points the status before sodium oxybate initiation and after optimum dose of sodium oxybate had been achieved, respectively. FU, follow-up is reported as median; IQR, interquartile range. The term combination treatment refers to stimulants or wake promoters and anti-cataplectic agents.

Sodium oxybate significantly reduced the ESS (ΔESS = 4.3 ± 4.4, p < 0.0001). The most common optimal dose of sodium oxybate was 9 g/night (31/90, 33.3%), but many patients were treated with lower doses (22.2% required 4.5 g/night, 22% 6 g/night, and 18% 7.5 g/night).

The improvement in ESS (ΔESS) correlated with higher final dose of the drug (r = 0.389, p < 0.001), but no correlation was found with age and BMI. A stepwise regression analysis was performed for predictors of ΔESS in the group of patients that did not discontinue sodium oxybate due to side effects or lack of efficacy. Age, BMI, gender, number of other concomitant drugs, reduction of other drug dosages, final sodium oxybate dose, and baseline ESS were added as independent variables. Patients who were sleepier at baseline were more likely to experience the greatest reduction in ESS (n = 61, F = 4.876, R = 3.23, adjusted R^2^ = 0.083, p = 0.033). No difference was found between genders with regard to age, BMI, baseline ESS, sodium oxybate final dose and ΔESS (p > 0.05).

A similar analysis was performed assessing the efficacy of sodium oxybate in controlling cataplexy, demonstrating a significant reduction in frequency of cataplectic events (Δcataplexy 21.8 ± 18.5 events/week; p < 0.0001). There was no correlation between age, BMI, final dose of sodium oxybate, and Δcataplexy. A stepwise regression analysis assessing predictors of Δcataplexy, using the same variables outlined above, demonstrated that only the baseline cataplexy frequency predicted reduction in cataplexy events (n = 61, F = 137.602, R = 0.898, adjusted R^2^ = 0.801, p < 0.0001). There was no difference between genders in the number of baseline cataplexy events or in Δcataplexy (p > 0.05). Sixty-one patients were successfully treated with sodium oxybate (61/90, 67.7%) and all patients except for one were on other drugs for narcolepsy. Twenty of those (32.8%) were on one or more stimulant medication, 37 (60.7%) on a combination of stimulant and anti-cataplectic agents, and three (4.9%) on an anti-cataplectic agent. The administration of sodium oxybate led to a reduction in dosage of other drugs in 56% (34/60) of patients, of which the majority remained on combined treatment (70.6%), but in 10% (6/60) all other drugs were successfully discontinued. No clear predictors of a reduction of other medications following initiation of sodium oxybate initiation could be identified. In five patients sodium oxybate was ineffective and discontinued.

### 6.2 Safety and tolerability

A total of 34 possible SEs associated with the administration of sodium oxybate were recorded from patients' medical records (Table 2). 55.6% (50/90) of our patients experienced a SE and in almost half of these cases (48%, 24/50) the drug had to be discontinued with subsequent resolution of the SE.

**Table 2:** Observed side effects in 90 patients with narcolepsy with cataplexy treated with sodium oxybate.

	n (%)
**General**	
Amenorrhea	1 (1.1%)
Appetite loss	6 (6.6%)
Asthenia	1 (1.1%)
Bad taste	6 (6.6%)
Facial swelling	1 (1.1%)
Pain	4 (4.4%)
Peripheral edema	1 (1.1%)
Sweating	3 (3.3%)
Urinary incontinence	2 (2.2%)
**Psychiatric**	
Anxiety	4 (4.4%)
Hallucinations	2 (2.2%)
Mood swings	14 (15.5%)
Psychosis	3 (3.3%)
Suicidal ideation	1 (1.1%)
**Cardiac**	
Hypertension	1 (1.1%)
Palpitations	1 (1.1%)
**Infections**	
Viral infection	1 (1.1%)
**Gastrointestinal**	
Diarrhea	1 (1.1%)
Dry mouth	1 (1.1%)
Faecal incontinence	1 (1.1%)
GERD	2 (2.2%)
Nausea	16 (17.7%)
Vomit	6 (6.6%)
**Neurological**	
Cramps	1 (1.1%)
Dizziness	9 (9.9%)
Headaches	7 (7.7%)
Impaired concentration	6 (6.6%)
Myoclonus	1 (1.1%)
Unresponsiveness	4 (4.4%)
**Sleep disorders**	
Enuresis	11 (12.2%)
Insomnia	2 (2.2%)
Sleep walking	7 (7.7%)
Worse cataplexy	1 (1.1%)
Worse OSA	3 (3.3%)

GERD: gastroesophageal reflex disease, OSA: obstructive sleep apnea.

The most common SEs observed were nausea (17.7%), mood swings (15.5%) and enuresis (12.2%). One patient developed hypertension and three patients with known obstructive sleep apnea (OSA) required re-titration to higher continuous positive airway (CPAP) pressures despite no change in BMI or other clinical variables.

The SEs that led to drug discontinuation were viral infection, psychosis, suicidal ideation, mood swings, anxiety, headaches, impaired concentration, dizziness, unresponsiveness, nausea, vomit, diarrhea, faecal and urinary incontinence, gastroesophageal reflex disease, appetite loss, sweating, facial swelling, bad taste, pain, enuresis, worse cataplexy, hypertension and palpitations (11 patients experienced more than one SE). Among these the most common SEs resulting in drug discontinuation were nausea (25%), headaches (20.8%), mood swings (16.7%) and dizziness (16.7%). Psychosis as a serious limiting SE was identified in three patients, aged 51, 63 and 69 years old respectively, one of whom also exhibited suicidal ideation. These three patients underwent formal psychiatric assessment. None of these three patients had any previous relevant medical history or suffered from any other co-morbidity. All three were on an anti-cataplectic agent, combined with dexamphetamine in two of them and with modafinil in one another. Of note, psychotic symptoms resolved after discontinuation of sodium oxybate in all three patients.

No significant correlation between total SE incidence and age, BMI, gender and final dose of sodium oxybate prescribed was identified, but analysis of individual SEs demonstrated age to be associated with risk of psychosis (r = 0.234, p = 0.027) and enuresis (r = 0.251, p = 0.017), and BMI with dizziness and bad taste (r = 0.279, p = 0.021 and r = 0.257, p = 0.034 respectively). No statistically significant prediction model was generated using as the dependent variable any SE incidence and as independent variables age, BMI, gender, optimum dose of sodium oxybate prescribed, number of other concomitant drugs, reduction of other drug dosages, baseline ESS, and ΔESS (p = 0.333).

For those SEs resulting in discontinuation, time to drug discontinuation was added as an independent variable. Correlation analysis revealed that SEs resulting in drug discontinuation tended to occur early after the initiation of sodium oxybate, and thus at lower doses of the drug (r = −0.240, p = 0.027 and r = −0.482, p < 0.001 respectively). The time to drug discontinuation due to limiting SEs had a median 12 months (interquartile range 3.7–22.0). With regards to specific SEs that led to drug discontinuation, several correlations were found, amongst which age was again associated with psychosis (r = 0.238, p = 0.028) and enuresis (r = 0.252, p = 0.020) but also with headaches (r = 0.251, p = 0.017) and BMI with dizziness (r = 0.291, p = 0.021) and bad taste (r = 0.266, p = 0.035). When binary forward stepwise model analysis was performed aiming to predict the occurrence of any limiting SE using as independent variables the factors from the previous binary regression analysis (age, BMI, gender, optimum dose of sodium oxybate prescribed, number of other concomitant drugs, reduction of other drug dosages, baseline ESS and ΔESS) plus the time to drug discontinuation, increasing age (OR = 1.060, 95% CI = 1.004–1.119, p = 0.036) and shorter time to drug discontinuation (OR = 0.924, 95% CI = 0.886–0.963, p < 0.001) were both found to be associated with an increased likelihood of a limiting SE to occur.

## 7 Discussion

Few studies have explored the efficacy and safety of sodium oxybate outside the strictly regulated environment of a prospective controlled trial. Our cohort had 3116 patient-months of drug exposure, to our knowledge the highest exposure time described in the literature to date. These results support previous findings regarding the efficacy and safety of the drug [10], [12], [13], [17], [18], [19], [20], [21]. It should be noted however that, owing to limitations of access to sodium oxybate in the United Kingdom, our cohort is likely to reflect a more severe or refractory phenotype than the cohorts in previous trials, with patients who have more severe EDS and more severe cataplexy, and who are refractory to traditional stimulants and anti-cataplectic agents.

The data presented highlight a number of salient points relevant to clinical practice. In this retrospective “real-life” analysis, sodium oxybate proved to be efficacious in terms of reduction observed in daytime sleepiness. Despite our patients having a higher baseline ESS, despite stimulant therapy, than in previous studies [10], [12], [13], [19], [22], the relative reduction in ESS observed in our study is comparable to those reported in the literature [10], [12], [13], [19], [21]. Furthermore, our data suggest that those patients who are sleepier or who have more severe cataplexy at baseline are the patients that would be expected to experience the most benefit. The impact of sodium oxybate on frequency of cataplexy events in our study was significant, but as expected, of a lower magnitude compared to other studies, since our cohort had already demonstrated itself to be refractory to traditional anti-cataplectic agents [10], [13], [22]. Whilst only 10% of patients achieved removal of other drugs for narcolepsy, approximately half of the patients that remained on sodium oxybate saw a lowering in their other drug dosages. The optimal maintenance dose could not be predicted by gender, BMI or other clinical factors.

In our cohort, a higher percentage of SEs leading to drug discontinuation was observed than in previous studies, perhaps reflecting the impact of co-administration of stimulants and anti-cataplectic agents, or the longer follow-up period. Over half of the patients (55.6%) experienced at least one SE, consistent with previous studies [17], [19], [21], but 26.6% were forced to stop the drug due to intolerable SEs. This is significantly higher than described in the literature (4.4%–9.4%) [10], [13], [17]. Again, this may be because our cohort consisted of patients with treatment refractory narcolepsy, with the majority already on other stimulants or/and anti-cataplectic agents. Additionally, a higher percentage of our patients received a higher optimized dose of 9 g/night (33%) than in other studies [10], [13], [17].

Nausea, mood swings and enuresis were the most frequent SEs, similar to, and at a comparable rate to, those reported in previous studies [10], [13], [17], [19], [21]. SEs did not appear to be dose-dependent or gender-related, but seemed more likely to occur in older patients, and were more likely to arise early in the treatment course and thus at lower doses. Our results also provide some indirect support to concerns that sodium oxybate may worsen sleep-disordered breathing, with three of our patients on CPAP requiring an increase in CPAP dose [23], [24]. This stresses the need to remain vigilant for the development or exacerbation of OSA after the initiation of sodium oxybate, particularly as it is more common in patients with narcolepsy [25].

## 8 Limitations

Patients and data in this cohort were retrospectively identified and collected from our clinical records, providing a realistic snapshot of the efficacy and safety of sodium oxybate in clinical practice. While drawing this sample from a single institution signifies the utilisation of a consistent approach in data collection, a pre-established side effects list would have offered a more standardised assessment. As with all retrospective studies, data captured from the medical records are less reliable than prospective data entry. Prospective multi-centre studies would therefore add credence to our findings.

## 9 Conclusion

Sodium oxybate is an efficacious and generally safe treatment of patients with narcolepsy and cataplexy in standard clinical practice. Significant SEs that lead to drug withdrawal are more common in older patients and tend to occur early after initiation of the drug.

## Disclosures

This research did not receive any specific grant from funding agencies in the public, commercial, or not-for-profit sectors.

Dr Drakatos reports no disclosures.

Dr Lykouras reports no disclosures.

Ms D'Ancona reports no disclosures.

Mr. Higgins reports no disclosures.

Dr Gildeh reports no disclosures.

Dr Macavei reports no disclosures.

Dr Rosenzweig reports no disclosures.

Dr Steier reports no disclosures.

Dr Muza reports no disclosures.

Prof. Williams reports previous fee from UCB Pharma as a speaker. Prof. Williams reports no conflicts of interest.

Dr Kent reports no disclosures.

Dr Leschziner reports speaker's fees and honoraria for advisory boards from UCB Pharma. Dr Leschziner reports no conflicts of interest.

## Authors' contributorship statement

PD: Analysis, writing, design, interpretation, concept and discussion; DL: Data acquisition, analysis, writing; GD, SH, NG, RM, IR, JS, RM, AJW: Analysis, writing, interpretation and discussion, BDK, GL: Concept, analysis, writing, interpretation and discussion.
